# Automatic Exudate Detection from Non-dilated Diabetic Retinopathy Retinal Images Using Fuzzy C-means Clustering

**DOI:** 10.3390/s90302148

**Published:** 2009-03-24

**Authors:** Akara Sopharak, Bunyarit Uyyanonvara, Sarah Barman

**Affiliations:** 1 Department of Information Technology, Sirindhorn International Institute of Technology, Thammasat University 131 Moo 5, Tiwanont Road, Bangkadi, Muang, Pathumthani 12000, Thailand; E-mails: akara@siit.tu.ac.th, bunyarit@siit.tu.ac.th; 2 Faculty of Computing, Information Systems and Mathematics, Kingston University Penrhyn Road, Kingston upon Thames, Surrey, KT1 2EE, UK; E-mail: s.barman@kingston.ac.uk

**Keywords:** Exudates, diabetic retinopathy, non-dilated retinal images, Fuzzy C-Means clustering

## Abstract

Exudates are the primary sign of Diabetic Retinopathy. Early detection can potentially reduce the risk of blindness. An automatic method to detect exudates from low-contrast digital images of retinopathy patients with non-dilated pupils using a Fuzzy C-Means (FCM) clustering is proposed. Contrast enhancement preprocessing is applied before four features, namely intensity, standard deviation on intensity, hue and a number of edge pixels, are extracted to supply as input parameters to coarse segmentation using FCM clustering method. The first result is then fine-tuned with morphological techniques. The detection results are validated by comparing with expert ophthalmologists’ hand-drawn ground-truths. Sensitivity, specificity, positive predictive value (PPV), positive likelihood ratio (PLR) and accuracy are used to evaluate overall performance. It is found that the proposed method detects exudates successfully with sensitivity, specificity, PPV, PLR and accuracy of 87.28%, 99.24%, 42.77%, 224.26 and 99.11%, respectively.

## 1. Introduction

Diabetic retinopathy eye diseases are the main cause of vision loss and their prevalence is set to continue rising [1]. The screening of diabetic patients for the development of diabetic retinopathy can potentially reduce the risk of blindness in these patients [2–6]. Early detection enables laser therapy to be performed to prevent or delay visual loss and may be used to encourage improvement in diabetic control. Current methods of detection and assessment of diabetic retinopathy are manual, expensive and require trained ophthalmologists. Exudates are one of the primary signs of diabetic retinopathy [7, 8]. Automatic exudates detection would be helpful for diabetic retinopathy screening process.

Gardner *et al*. proposed an automatic detection of diabetic retinopathy using an artificial neural network. The exudates are identified from grey level images and the fundus image is analyzed using a back propagation neural network. The classification of a 20×20 region is used rather than a pixel-level classification [9]. Sinthanayothin *et al*. reported the result of an automated detection of diabetic retinopathy on digital fundus images by a Recursive Region Growing Segmentation (RRGS) algorithm on a 10×10 window [10]. In the preprocessing step, adaptive, local, contrast enhancement is applied. The optic disc, blood vessels and fovea detection are also localized [6]. Wang *et al*. used color features on a Bayesian statistical classifier to classify each pixel into lesion or non-lesion classes [11]. Phillips *et al*. have applied a thresholding technique based on the selection of regions to detect exudates. A patch of size 256 × 192 pixels is selected over the area of interest. Global thresholding is used to detect the large exudates, while local thresholding is used to detect the lower intensity exudates [12]. Huiqi Li *et al*. proposed an exudate extraction technique by using a combination of region growing and edge detection techniques. The optic disc is also detected by principal component analysis (PCA). The shape of the optic disc is detected using a modified active shape model [13]. Sanchez *et al*. combined color and sharp edge features to detect the exudates. The yellowish objects are detected first; the objects in the image with sharp edges are then detected using Kirsch’s mask and different rotations of it on the green component. The combination of results of yellowish objects with sharp edges is used to determine the exudates [5]. Hsu *et al*. presented a domain knowledge based approach to detect exudates. A median filter is used to compute an intensity difference map. Dynamic clustering is then used to determine lesion clusters. Finally domain knowledge is applied to identify true exudates [2]. Usher *et al*. detected the candidate exudates region by using a combination of RRGS and adaptive intensity thresholding [14]. Goh *et al*. used the minimum distance discriminant to detect the exudates. The spectrum feature center of exudates and background are computed and then the distance from each pixel to class center is calculated. The pixel is classified as exudate if it falls within the minimum distance [15]. Ege *et al*. used a median filter to remove noise. Bright lesions and dark lesions are separated by thresholding. A region growing algorithm is used to locate exudates. Bayesian, Mahalanobis and K-Nearest Neighbor classifier were tested. From these experiments, the Mahalanobis classifier was shown to yield the best results [16]. Walter *et al*. detected exudates using grey level variation and contours determined by means of morphological reconstruction techniques [17]. Niemeijer *et al*. have proposed a machine learning-based to detect exudates [18].

The Fuzzy C-Means (FCM) clustering is a well-known clustering technique for image segmentation. It was developed by Dunn [19] and improved by Bezdek [20]. It has also been used in retinal image segmentation [3, 21–24]. Osareh *et al*. used color normalization and a local contrast enhancement in a pre-processing step. The color retinal images are segmented using Fuzzy C-Means (FCM) clustering and the segmented regions are classified into two disjoint classes – exudate and nonexudate patches – using a neural network [3, 21]. The comparative exudate classification using Support Vector Machines (SVM) and neural networks was also applied. They showed that SVM are more practical than the other approaches [23]. Xiaohui Zhang and Chutatape Opas used local contrast enhancement preprocessing and Improved FCM (IFCM) in Luv color space to segment candidate bright lesion areas. A hierarchical Support Vector Machines (SVM) classification structure was applied to classify bright non-lesion areas, exudates and cotton wool spots [24].

Many techniques have been performed for exudate detection, but they have limitations. Poor quality images affect the separation result of bright and dark lesions using thresholding and exudate feature extraction using the RRGS algorithm, while other classification techniques require intensive computing power for training and classification. Furthermore, based on experimental work report in the previous work, most of techniques mention above worked on images taken when the patient had dilated pupils. Good quality retinal images with large fields that are clear enough to show retinal detail are required to achieve good algorithm performance. Low quality images (non-uniform illumination, low contrast, blurred or faint images) do not give good results even when enhancement processes are included. The examination time and effect on the patient could be reduced if the automated system could succeed on non-dilated pupils.

## 2. Materials and Methods

Forty digital retinal images of patient are obtained from a KOWA-7 non-mydriatic retinal camera with a 45° field of view. The images were stored in a JPEG image format (.jpg) files using the lowest compression rates. The image size is 500 × 752 pixels at 24 bit.

### 2.1. Exudate detection

Exudates can be identified on the ophthalmoscope as areas with hard white or yellowish colors with varying sizes, shapes and locations. They normally appear near the leaking capillaries within the retina. The main cause of exudates are proteins and lipids leaking from the blood into the retina via damaged blood vessels [3, 8]. This part of the paper describes how FCM clustering is use and how the features are selected and used.

### 2.2 Coarse Segmentation using Fuzzy C-Means Clustering

FCM clustering is an overlapping clustering algorithm, where each point may belong to two or more clusters with different degrees of membership. The features (discussed in Section 2.3) with close similarity in an image are grouped into the same cluster. The similarity is defined by the distance of the features vector to the cluster centers. Euclidean distance is used to measure this distance and data will be associated to an appropriate membership value [24, 29, 30]. The cluster center is updated until the difference between adjacent objective function, as displayed in Equation 1 is close to zero or practically less than a predefined small constant:

(1) $$J_{m}=\sum_{i=1}^{M}\sum_{j=1}^{C}u_{\mathrm{ij}}^{m}{\left\|x_{i}-c_{j}\right\|}^{2}$$

where *m* is an exponential weighting function that controls the fuzziness of the membership function, it is set to 2 by Bezdek [20]. *M* is number of features. *C* is number of clusters. *u_ij_* is the degree of membership of *x_i_* in the cluster *j*, *x_i_* is the *i*th of d-dimensional measured data, *c_j_* is the d-dimension center of the cluster, and ||*|| is any norm expressing the similarity between any measured feature and the center.

Fuzzy partitioning is carried out through an iterative optimization of the objective function shown above, with the update of membership *u_ij_* and the cluster centers *c_j_* by Equations 2 and 3:

(2) $$u_{\mathrm{ij}}=\frac{1}{\sum_{k=1}^{c}{\left(\frac{\left\|x_{i}-c_{j}\right\|}{\left\|x_{i}-c_{k}\right\|}\right)}^{\frac{2}{m-1}}}$$

(3) $$c_{j}=\frac{\sum_{i=1}^{M}u_{\mathrm{ij}}^{m}x_{i}}{\sum_{i=1}^{M}u_{\mathrm{ij}}^{m}}$$

The iteration will stop when Equation 4 is satisfied:

(4) $${\text{max}}_{\mathrm{ij}}\left\{\left|u_{\mathrm{ij}}^{\left(k+1\right)}-u_{\mathrm{ij}}^{\left(k\right)}\right|\right\}<\varepsilon$$

where ε is a termination criterion, 0.00001 for our case. *k* is the iteration number, it is set to a maximum of 200 for our case. This procedure converges to a local minimum or a saddle point of *J_m_*.

The input to the FCM algorithm is a set of features. Rhe algorithm is composed of the following steps:

Step 1: Initialize the fuzzy partition matrix *U = [u_ij_]* (*U^(0)^*) by generating random numbers in the range 0 to 1 subject to Equation 5:
  (5) $$\sum_{i=1}^{M}\sum_{j=1}^{C}u_{\mathrm{ij}}=1$$
Step 2: At *k*-step: calculate the centers vectors *C^(K)^=[c_j_]* with *U^(K)^* according to Equation 3.
Step 3: Update the fuzzy partition matrix *U^(K)^*, *U^(K+1)^* by the new computed *u_ij_* according to Equation 2.
Step 4: Compute the objective function according to Equation 1. If the difference between adjacent values of the objective function is less than termination criterion (ε) then stop the iteration; otherwise return to step 2.

The output from FCM clustering is a list of cluster centers and *n* membership-grades for each pixel, where *n* is a number of desired clusters. A pixel will be assigned to the cluster with highest membership-grade.

### 2.3 Feature selection

We asked ophthalmologists how they identify exudates in an image so that our feature selection would reflex ophthalmologists’ expertise. We found that color, shape and texture are among those top features they look at. To differentiate exudate pixels from non-exudates pixels, we attempt to mimic ophthalmologist expertise by extracting these relevant and significant features. Four features are empirically selected and used as input for FCM clustering. They are intensity value after preprocessing, standard deviation of intensity, hue and number of edge pixels from an edge image. The reasons for the features selection and their details are explained in this section.

**Figure 1.**
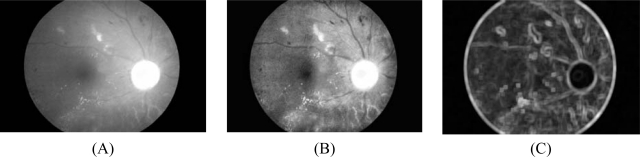
Pre-processing result. (A) Original I band. (B) I band after pre-processing. (C) Standard deviation of (A).

**Figure 2.**
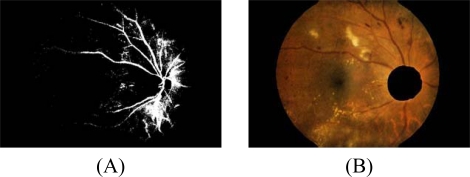
Blood vessel and optic disc detection. (A) Blood vessel detected from decorrelation stretch image. (B) Optic disc area eliminated from the contrast enhanced image.

**Figure 3.**
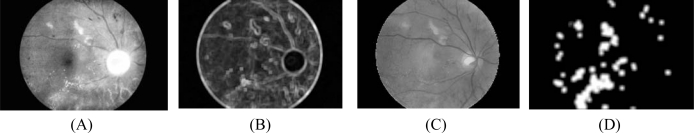
Input features for FCM clustering of image1. (A) Intensity image after preprocessing. (B) Standard deviation of intensity image. (C) Hue image. (D) Image of edge pixels.

1. Intensity image after pre-processing (*I_CLAHE_*) is selected as one of the classification features because exudate pixels can usually be distinguished from normal pixels by their intensity. Firstly, the Red, Green and Blue (RGB) space in the original image is transformed to Hue, Saturation and Intensity (HSI) space. A median filtering operation is then applied on the I (intensity) band to reduce noise before a Contrast-Limited Adaptive Histogram Equalization (CLAHE) is applied for contrast enhancement [23]. The original intensity band image and intensity band after preprocessing are shown in Figures 1A and 1B, respectively.
2. Standard deviation of *I_CLAHE_* is also chosen as an input parameter because distribution measurement of the pixel values would differentiate exudate area from the others since standard deviation shows the main characterization of the closely distributed cluster of exudates. The standard deviation of the intensity bands after preprocessing is shown in Fig. 1C. Standard deviation is defined in Equation 6:
  (6) $$\mathrm{Std}(x)=\frac{1}{N-1}\cdot \sum_{i\in W(x)}{\left(I_{\mathrm{CLAHE}}(i)-\mu_{I_{\mathrm{CLAHE}}}(x)\right)}^{2}$$
  where *x* is a set of all pixels in a sub-window *W(x)*, *N* is a number of pixels in *W(x)*, μ_*I*_*CLAHE*__ *(x)* is mean value of *I_CLAHE_(i)* and *i*∈*W(x*). A window size of 15 × 15 pixels was used in this step.
3. Hue, also extracted from HSI space, is the third feature selected for input to FCM clustering because hue components make up chrominance or color information. From visual inspection, exudates appear differently in a yellowish or white color.
4. Normally exudates gather together in small clusters so they tend to have many edge pixels around the area. A number of edge pixels is also selected as our last feature to FCM clustering. However, during this feature extraction, we remove some irrelevant edge pixels, as described:
  4.1 For fast edge detection, a Sobel edge operator with a mask size of 3×3 pixels is used to compute the gradient magnitude.
  4.2 The result from the previous step is then thresholded by a fixed and low value in order to get most of the edge pixels.
  4.3 However, some of the resulting edge pixels from the previous step do not represent the edge of the exudates. Some of them are part of vessel’s edge and these vessel edge pixels need to be removed before proceeding to the next step. Quick and approximate blood vessel detection is achieved by using a decorrelation stretch on the Red band. The decorrelation stretching is a process used to enhance or stretch the color differences found in a color image. Contrast exaggeration is used to expand the range of intensities of highly correlated images [24, 25]. Blood vessels can be detected by thresholding this result and the detection result are shown in Fig. 2A.
  4.4 Some exudates are soft exudates which cannot be detected by a strong edge. High-value red pixels selected from the decorrelation stretch image are chosen and added to the result from the previous step because the soft exudates normally appear red. However, red pixels which belong to the optic disc, which also appear red, have to be removed first.
  The optic disc is quickly detected by using an entropy feature on *I_CLAHE_*. The entropy is a statistic measurement of randomness that can be used to characterize the texture of the input image. The optic disc which is normally smooth appears in relatively low intensity in Entropy space. The resulting image is thresholded at an automatically selected grey level, using the Otsu algorithm [26]. Normally, the optic disc can be easily identified as the largest area. However, in some cases, such as the appearance of huge exudates in the image, there might be some areas in the image which are larger than the optic disc. Because the shape of optic disc is round, therefore the optic disc region selection process needs to be made specific to the largest one among the regions whose shapes have compactness, as calculate by Equation 7, close to one. To ensure that all the neighbouring pixels of the thresholded result are also included in the candidate region, a binary dilation operator is also applied. For this step, a flat disc-shaped structuring element with a fixed radius of 11 is used. An example result of an image with all the optic disc area masked out is shown in Figure 2B.
  4.5 A number of neighboring white pixels of the resulting image from the process 4.1 – 4.4 is counted using a window size of 17 × 17 to form our final feature, namely an image of the number of edge pixels as shown in Figure 3D:
    (7) $$\mathrm{Compactness}=4\pi (\mathrm{area})/{\left(\mathrm{perimeter}\right)}^{2}$$
    where *area* is the number of pixels in the region and *perimeter* is the total number of pixels around the boundary of each region.

There are many parameters used in this experiment. They are, namely, window size in standard deviation, window size in fast edge detection using Sobel operator, the size of structuring element used for dilation operation in optic disc detection, window size used for find a number of edge pixel and a number of cluster. They are varied and tested empirically in order to assess the algorithm performance and parameters which give highest accuracy are chosen. Note that this manual parameters adjustment is a form of algorithm training and can significantly influence final evaluation, if the data is not sufficiently large, by introducing a positive bias. Example result of the four features is shown in Figure 3. These four features will be used in the segmentation process as described in the next section.

### 2.4 Fine Segmentation using Morphological Reconstruction

**Figure 4.**
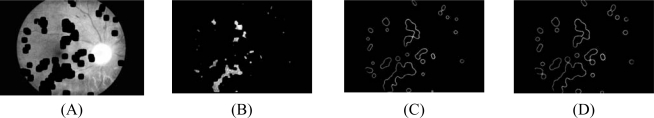

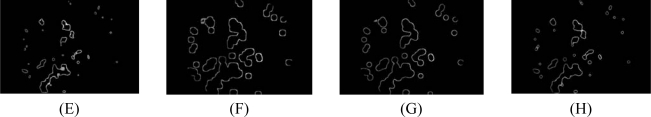
FCM clustering results with n=8. (A) Cluster 1. (B) Cluster 2. (C) Cluster 3. (D) Cluster 4. (E) Cluster 5. (F) Cluster 6. (G) Cluster 7. (H) Cluster 8.

The FCM clustering algorithm is applied to forty test images to get a result of eight clusters (*n*=8) for each image; the result is shown in Figure 4.

The result from the previous section is a rough estimation of the exudates. In order to get a better result, a fine segmentation using morphological reconstruction is applied in this step. Morphology reconstruction is a part of morphological image processing. Morphological reconstruction is based on dilation on two images, a marker and a mask.

**Figure 5.**
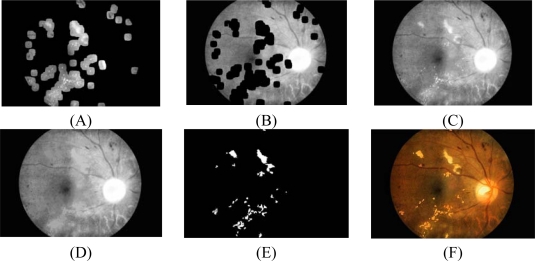
Exudates detection (A) Candidate areas after using FCM clustering. (B) Marker image. (C) Mask image. (D) Reconstructed image. (E) Difference image. (F) Result superimposed on the original image.

The important cluster obtained from the previous steps is the cluster which contains most of the original image information but all exudate areas are missing, as displayed in Figure 4A. The term first cluster will be used throughout the text to represent this cluster even though it might not belong to the first cluster of clustering result. The exudate pixels can be obtained by subtracting this first cluster with the original intensity image, as displayed in Figure 5A. The first cluster is again used as a marker while the original intensity image is used as a mask. The morphological reconstruction by dilation is then applied on the previous overlaid image. Dilations of the marker image under the mask image are repeated until the contour of the marker image fits under the mask image. The result is displayed in Figure 5D.

Final result is obtained by applying a threshold operation at automatically selected grey levels to the difference between the original image and the reconstructed image. The result image is shown in Figure 5E and Figure 5F shows the result superimposed on the original image.

### 2.5 Performance measurement

As a simple baseline for comparison, nearest neighbor classifier with Euclidean distance is used. The nearest neighbour classifier simply classifies a test instance with the class of the nearest training instance according to some distance measure. Performance of each parameter is measured by comparing the detection results with ophthalmologists’ hand-drawn ground truth. Nine performance measurements, namely, true positive (TP, a number of exudates pixels correctly detected), false positive (FP, a number of non-exudate pixels which are detected wrongly as exudate pixels), false negative (FN, a number of exudate pixels that are not detected), true negative (TN, a number of nonexudates pixels which are correctly identified as non-exudate pixels), sensitivity, specificity, positive predictive value (PPV), positive likelihood ratio (PLR) and accuracy are calculated [31,32]. Equations 8, 9, 10, 11 and 12 show the computation of sensitivity, specificity, PPV, PLR and accuracy, respectively:

(8) $$\mathrm{Sensitivity}=\frac{\mathrm{TP}}{\mathrm{TP}+\mathrm{FN}}$$

(9) $$\mathrm{Specificity}=\frac{\mathrm{TN}}{\mathrm{TN}+\mathrm{FP}}$$

(10) $$\mathrm{PPV}=\frac{\mathrm{TP}}{\mathrm{TP}+\mathrm{FP}}$$

(11) $$\mathrm{PLR}=\frac{\mathrm{TP}/(\mathrm{TP}+\mathrm{FN})}{\mathrm{FP}/(\mathrm{FP}+\mathrm{TN})}=\frac{\mathrm{Sensitivity}}{1-\mathrm{Specificity}}$$

(12) $$\mathrm{Accuracy}=\frac{\mathrm{TP}+\mathrm{TN}}{\mathrm{TP}+\mathrm{FP}+\mathrm{FN}+\mathrm{TN}}$$

## 3. Results

Forty images were tested on an AMD Athlon 1.25 GHz PC using the MATLAB platform. Each image took approximately 18 minutes for FCM clustering and another three minutes for morphological reconstruction. The result from the coarse segmentation was used as input to the fine segmentation using morphological reconstruction.

**Figure 6.**
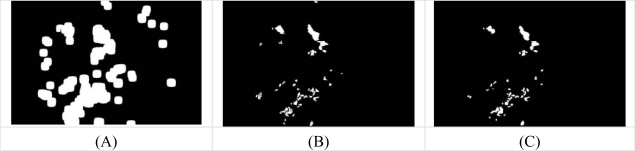
Comparison of exudates detection. (A) Coarse segmentation using FCM clustering. (B) Fine segmentation using morphological reconstruction (C) Ground truth image.

**Figure 7.**
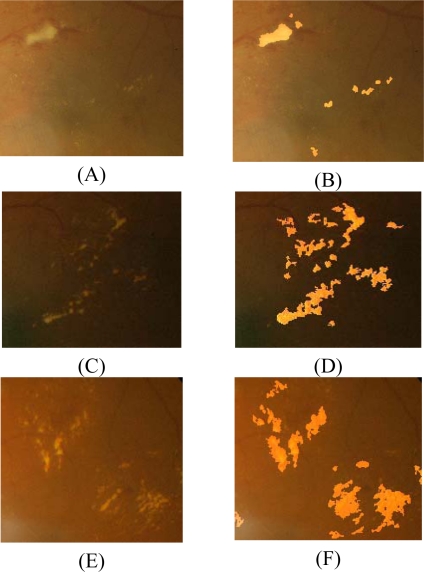
Exudates detection on low contrast images (A), (C) and (E) are original images, (B), (D) and (F) are detected exudates superimposed on original images of (A), (C) and (E) respectively.

After fine segmentation, most of the classified exudate regions are true exudate pixels, which gives a smaller true positive value; however, it also reduces the false positive value because misclassification of non-exudate pixels is also lower. Figure 6 displays the comparison of exudate detection from the first cluster resulting from coarse segmentation and result of FCM clustering followed by morphological reconstruction and a ground-truth image. Three examples of exudates detection result of FCM clustering followed by morphological reconstruction are shown in Figure 7.

The performance of our technique was evaluated quantitatively by comparing the result of extractions with ophthalmologists’ hand-drawn ground-truth images. Ten examples of detailed results of performance measurement using FCM clustering followed by morphological reconstruction are displayed in Table 1. The sensitivity, specificity, PPV, PLR and accuracy of validation results are shown in Table 2.

From experimental results, if FCM clustering is the only technique used; it gives a high true positive value but also a high false positive value. However, the PPV and PLR values are low. Using FCM clustering followed by morphological reconstruction, we have higher accuracy with a lower false positive value. Comparing with baseline algorithm, the results indicate that the FCM clustering followed by morphological performs better in PPV, PLR, accuracy than nearest neighbor.

## 4. Discussion and Conclusions

In this paper, we have investigated and proposed methods to automatically extract exudates from images taken from diabetic patients with non-dilated pupils. The work is based on the FCM clustering segmentation and morphological techniques. Four input features based on the characteristics of exudates, namely intensity, standard deviation, hue and number of edge pixels, are selected. Blood vessels and optic disc pixels are also removed from the fourth feature in order to prevent misclassification. The performance of the algorithm is measured against ophthalmologists’ hand-drawn ground-truth. Sensitivity, specificity, PPV and PLR are used as the performance measurement of exudate detection because they combine true positive and false positive rates. Accuracy values are also used to evaluate the system.

The result shows that PPV, PLR and accuracy values increase when the FCM clustering technique is combined with morphological technique. If any applications need to detect maximum number of exudate pixels or require more execution speed, the FCM clustering technique could be used in isolation. However, if the applications require higher accuracy, the FCM clustering combined with the morphological technique should be chosen.

There are some incorrect exudate detections which are caused by the artifacts that are similar to exudates, artifacts from noise in the image acquisition process, the exudates that are proximate to blood vessels or exudates that appear very faint. These missing faint exudates may have not affected the sensitivity much since even human experts are not sure about some ambiguous regions. However, the performance of the algorithm can be improved if these set of low-contrast exudates can be detected. This system intends to help ophthalmologists in diabetic retinopathy screening process to detect symptoms faster and more easily. This is not a final-result application but it can be a preliminary diagnosis tool or decision support system for ophthalmologists. Human ophthalmologists are still needed for the cases where detection results are not very obvious.

One main weakness of the algorithm arises from the fact that the algorithm depends on other tasks, namely, the detection of optic disc, and vessel removal. The result of exudate detection depends on the success of these methods. Future work will address improvement of the performance of this system by improving the robustness of optic disc and blood vessel detection and finding more specific characteristics of exudates which could distinguish them more effectively. A supervised clustering method might be used in order to obtain better result.

## Figures and Tables

**Table 1.** The example results of exudates detection from FCM clustering followed by morphological operator

**24-bit images**	**TP**	**FP**	**FN**	**TN**	**Sensitivity (%)**	**Specificity (%)**	**PPV (%)**	**PLR**	**Accuracy (%)**
Image1	4898	1677	741	368684	86.86	99.55	74.49	191.83	99.36
Image2	1019	350	61	374570	94.35	99.91	74.43	1010.70	99.89
Image3	81	3548	27	372344	75.00	99.06	2.23	79.46	99.05
Image4	838	2294	227	372641	78.69	99.39	26.76	128.60	99.33
Image5	1247	4978	67	369708	94.90	98.67	20.03	71.43	98.66
Image6	1479	2905	204	371412	87.88	99.22	33.74	113.23	99.17
Image7	126	2445	23	373406	84.56	99.35	4.90	129.99	99.34
Image8	1234	2734	525	371507	70.15	99.27	31.10	96.03	99.13
Image9	381	322	38	375259	90.93	99.91	54.20	1060.62	99.90
Image10	424	1729	76	373771	84.80	99.54	19.69	184.17	99.52

**Table 2.** Comparison of average result from FCM clustering only, FCM clustering followed by morphological reconstruction and nearest neighbor

**Method**	**Sensitivity (%)**	**Specificity (%)**	**PPV (%)**	**PLR**	**Accuracy (%)**
FCM clustering	97.2	85.4	5.9	7.9	85.6
FCM clustering and morphological	87.2	99.2	42.7	224.2	99.1
Nearest neighbor	90.4	96.6	28.6	6.2	96.5

## References

[1] Olson J.A., Strachana F.M., Hipwell J.H. (2003). A comparative evaluation of digital imaging, retinal photography and optometrist examination in screening for diabetic retinopathy. Diabet. Med.

[2] Hsu W., Pallawala P.M.D.S., Lee Mong Li, Eong Kah-Guan Au The Role of Domain Knowledge in the Detection of Retinal Hard Exudates.

[3] Osareh A., Mirmehdi M., Thomas B., Markham R. (2003). Automated Identification of Diabetic Retinal Exudates in Digital Colour Images. Br. J. Ophthalmol.

[4] Paisan R., Nattapon W., Pattanaporn S., Ekchai P., Montip T. (2005). Screning for Diabetic Retinopathy in Rural Area Using Single-Field, Digital Fundus Images. J. Med. Assoc. Thailand.

[5] Sanchez C.I., Hornero R., Lopez M.I., Poza J. Retinal Image Analysis to Detect and Quantify Lesions Associated with Diabetic Retinopathy.

[6] Sinthanayothin C., Boyce J.F., Cook H.L., Williamson T.H. (1999). Automated Localization of the Optic Disc, Fovea, and Retinal Blood Vessels from Digital Colour Fundus Images. Br. J. Ophthalmol.

[7] Bjorvis S., Johansen M.A., Fossen K. (2002). An economic analysis of screening for diabetic retinophathy. J. Telemed. Telecare.

[8] Feman S.S., Leonard-Martin T.C., Andrews J.S. (1995). A quantitative system to evaluate diabetic retinopathy from fundus photographs. Invest. Ophthalmol. Vis. Sci.

[9] Gardner G.G., Keating D, Williamson T.H., Elliot A.T. (1996). Automatic Detection of Diabetic Retinopathy using an Artificial Neural Network: a Screening Tool. Br. J. Ophthalmol.

[10] Sinthanayothin C., Boyce J.F., Williamson T.H., Cook H.L. (2002). Automated Detection of Diabetic Retinopathy on Digital Fundus Image. Diabet. Med.

[11] Wang, Hsu H., Goh W., Lee K.G. An Effective Approach to Detect Lesions in Color Retinal Images.

[12] Phillips R.P., Forrester J., Sharp P. (1993). Automated detection and quantification of retinal exudates. Graefe Arch Clin. Exp. Ophthalmol.

[13] Huiqi L., Chutatape O. (2003). A model-based approach for automated feature extraction in fundus images. Internat. Conf. on Computer Vision (ICCV).

[14] Usher D., Dumskyj M., Himaga M., Williamson T.H., Nussey S., Boyce J. (2004). Automated detection of diabetic retinopathy in digital retinal images: a tool for diabetic retinopathy screening. Diabet. Med.

[15] Goh K.G., Hsu W., Lee Li, Wang H., Krzysztof J.C. (2001). ADRIS: an Automatic Diabetic Retinal Image Screening system. Medical data mining and knowledge discovery.

[16] Ege B.M., Hejlese O.K., Larsen O.V., Moller K., Jennings B., Kerr D., Cavan D.A. (2000). Screening for diabetic retinopathy using computer based image analysis and statistical classification. Comput. Meth. Programs Biomed.

[17] Walter T., Klein J.C., Massin P., Erginay A. (2002). A Contribution of Image Processing to the Diagnosis of Diabetic Retinopathy-Detection of Exudates in Colour Fundus Images of the Human Retina. IEEE Transactions on Medical Imaging.

[18] Niemeijer M., Ginneken B.V., Russell S.R., Suttorp-Schulten M.S.A., Abramoff M.D. (2007). Automated detection and differentiation of drusen, exudates, and cotton-wool spots in digital color fundus photographs for diabetic retinopathy diagnosis. Invest. Ophthalmol. Vis. Sci.

[19] Dunn J.C. (1973). A Fuzzy Relative of the ISODATA Process and Its Use in Detecting Compact Well-Seperated Clusters. J. Cyber.

[20] Bezdek J.C. (1981). Pattern Recognition with Fuzzy Objective Function Algorithms.

[21] Osareh A., Mirmehdi M., Thomas B., Markham R., Claridge E., Bamber J. (2001). Automatic recognition of exudative maculopathy using fuzzy c-means clustering and neural networks. Medical Image Understanding Analysis.

[22] Osareh A., Mirmehdi M., Thomas B., Markham R. (2002). Classification and Localisation of Diabetic-Related Eye Disease. Internat. European Conf. on Computer Vision.

[23] Osareh A., Mirmehdi M., Thomas B., Markham R. (2002). Comparative Exudate Classification using Support Vector Machines and Neural Networks. Internat. Conf. on Medical Image Computing and Computer-Assisted Intervention.

[24] Zhang Xiaohui, Chutatape O. Top-down and bottom-up strategies in lesion detection of background diabetic retinopathy.

[25] Gonzalez R.C., Woods R.E. (2002). Digital image processing.

[26] Gillespie A.R., Kahle A.B., Walker R.E. (1986). Color Enhancement of Highly Correlated Images. I. Decorrelation and HIS Contrast Stretch. Remote Sens. Environ.

[27] Phung Son Lam, Abdesselam Bouzerdoum, Douglas Chai (2005). Skin Segmentation Using Color Pixel Classification: Analysis and Comparison. IEEE Transactions on Pattern Analysis and Machine Intelligence.

[28] Otsu N. (1979). A threshold selection method from gray-level histograms. IEEE Trans. on Syst. Man. and Cybern.

[29] Musa H., Musa Alci. (2005). Reliability analysis of microarray data using fuzzy c-means and normal mixture modeling based classification methods. Bioinformatics.

[30] Wang X.Y., Garibaldi J., Ozen T. Application of The Fuzzy C-Means clustering Method on the Analysis of non Pre-processed FTIR Data for Cancer Diagnosis.

[31] Attia John (2003). Moving beyond sensitivity and specificity: using likelihood ratios to help interpret diagnostic tests. Austral. Prescrib.

[32] Kallergi Maria, Costaridou L. (2005). Evaluation Strategies for Medical-Image Analysis and Processing Methodologies. Medical image analysis methods: the electrical engineering and applied signal processing series.

